# Evaluation of the Phytochemical Composition, Antioxidant Activity, and Enzyme Inhibitory Potential of *Salvia heldreichiana* Within the Framework of Molecular Docking and CAVER Tunnel Analysis

**DOI:** 10.1002/open.70240

**Published:** 2026-06-01

**Authors:** Erdi Can Aytar, Esin Çolak, Kaan Bedirhan Kahveci, Abidin Gümrükçüoğlu, Süleyman Doğu, Betül Aydın

**Affiliations:** ^1^ Faculty of Agriculture Department of Horticulture Usak University Usak Türkiye; ^2^ Faculty of Science Department of Biology Gazi University Ankara Türkiye; ^3^ Medicinal‐Aromatic Plants Application and Research Center Artvin Çoruh University Artvin Türkiye; ^4^ Faculty of Medicine Department of Medical Biochemistry Necmettin Erbakan University Konya Türkiye; ^5^ Meram Vocational School Konya Necmettin Erbakan University Türkiye

**Keywords:** acetylcholinesterase inhibition, CAVER tunnel analysis, molecular docking, *Salvia heldreichiana*

## Abstract

Medicinal plants are widely used in traditional medicine, and *Salvia* species hold an important place in Turkish folk medicine. This study comprehensively evaluated the phytochemical composition, antioxidant potential, enzyme inhibitory activity, molecular docking, and CAVER tunnel properties of *Salvia heldreichiana*. HPLC analysis identified 4‐hydroxybenzoic acid, rosmarinic acid, p‐coumaric acid, and chrysin as the major bioactive constituents. The extract exhibited high total phenolic content and notable DPPH radical scavenging activity, indicating strong antioxidant potential. GC–MS analysis showed that the volatile fraction was mainly composed of 1,8‐cineole, α‐pinene, and p‐cymene. The methanolic extract also displayed measurable inhibitory activity against acetylcholinesterase and tyrosinase, suggesting potential relevance for neuroprotective and dermatological applications. Molecular docking results demonstrated that rosmarinic acid and chrysin showed strong binding affinities toward the selected protein targets, including 1ACJ, 2Y9X, and 3NVY. In addition, CAVER analysis revealed continuous and structurally feasible tunnels connecting active sites with the protein surface, supporting the plausibility of ligand migration. Overall, the findings suggest that *S. heldreichiana*, owing to its rich phenolic profile and bioactive properties, may represent a promising natural source for supportive applications against oxidative stress–related and enzyme‐associated disorders.

## Graphical abstract

Methanolic extract of *Salvia heldreichiana* is characterized by HPLC and GC–MS analyses and evaluated for antioxidant and anti‐Alzheimer activities. Major phenolic compounds are identified, while molecular docking analysis supports the interaction of bioactive constituents with target enzymes, highlighting the therapeutic potential of *S. heldreichiana* as a natural neuroprotective source.

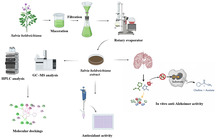

## 1 Introduction

Free radicals are highly reactive and unstable atoms or molecules containing one or more unpaired electrons [1]. Among these, reactive oxygen species (ROS) and reactive nitrogen species (RNS) are considered the most biologically significant groups. They are continuously generated through normal cellular metabolism as well as exposure to environmental factors such as ultraviolet radiation, cigarette smoke, air pollution, and industrial chemicals [2].

Neurodegenerative diseases are progressive disorders that predominantly occur in advanced age and are characterized by the gradual loss of neuronal structure and function [3, 4]. Alzheimer's disease, Parkinson's disease, Huntington's disease, and amyotrophic lateral sclerosis are among the most common neurodegenerative disorders. Due to its high oxygen consumption and limited antioxidant defense system, the brain is especially susceptible to oxidative damage, making oxidative stress a major factor in neurodegeneration [5]. Therefore, natural antioxidants have attracted considerable scientific interest because of their potential to neutralize free radicals and protect neuronal integrity. Increasing evidence suggests that dietary antioxidants may help slow the progression of neurodegenerative diseases such as Alzheimer's and Parkinson's disease [6]. In Alzheimer's disease, the abnormal accumulation of amyloid‐β (Aβ) peptides contributes to neuronal dysfunction, oxidative stress, inflammation, and cognitive decline, ultimately impairing learning and memory functions [7].

Currently, the pharmacological treatment of Alzheimer's disease is mainly based on acetylcholinesterase (AChE) inhibitors and N‐methyl‐D‐aspartate (NMDA) receptor antagonists. Commonly used AChE inhibitors such as donepezil, galantamine, and rivastigmine enhance cholinergic neurotransmission by preventing the enzymatic degradation of acetylcholine in the brain [8]. Donepezil and galantamine act as reversible AChE inhibitors, whereas rivastigmine inhibits both acetylcholinesterase and butyrylcholinesterase. Although these drugs are widely used in the symptomatic management of Alzheimer's disease, their therapeutic effectiveness may decrease over time due to the progressive loss of cholinergic neurons. Donepezil is approved for all stages of the disease, while rivastigmine and galantamine are generally prescribed for mild to moderate Alzheimer's disease [9].

Interest in plant‐derived antioxidants has increased considerably in recent years because of their potential protective effects against oxidative stress–related aging and chronic diseases. Medicinal plants are regarded as important natural sources of antioxidant compounds, particularly due to their rich phenolic and flavonoid contents [10]. Clinical studies investigating aromatic plant extracts in Alzheimer's disease have reported promising findings. For example, the topical application of *Melissa officinalis* essential oil was associated with reduced agitation levels and improved quality of life in Alzheimer's patients [11]. Similarly, massage therapy with *M. officinalis* essential oil improved agitation and neuropsychiatric symptoms in a placebo‐controlled clinical trial [12]. In addition, *Lavandula angustifolia* essential oil has been reported to promote calming effects and improve sleep quality in dementia patient [13]. Another study demonstrated that regular inhalation of essential oils contributed to improvements in cognitive function and orientation in individuals with Alzheimer's disease [14].

Lamiaceae family's greatest diversity is found in Central and South America, accounting for around 500 species, followed by the Mediterranean and Central Asian regions with nearly 250 species. East Asia also constitutes an important diversity center, hosting more than 90 species [15]. The name *Salvia* is derived from the Latin verb *salvare*, meaning “to heal” or “to preserve health.” This etymological origin reflects the long‐standing association of the plant with therapeutic properties and explains its extensive use in traditional medicinal practices throughout history [16]. Studies reported in the literature indicate that compounds isolated from various *Salvia* species exhibit significant free radical scavenging activity [17, 18]. In addition, these species have been shown to possess antibacterial and antifungal properties and demonstrate cytotoxic effects against certain cell lines activities [18]. This broad spectrum of biological activities highlights *Salvia* as a valuable source of bioactive compounds and underscores its growing importance in the development of novel medicinal and industry‐oriented natural products.

Türkiye is considered one of the major global centers of diversity for the genus *Salvia*. Approximately 92 *Salvia* taxa have been documented in Türkiye, nearly half of which are endemic. This high level of endemism highlights the necessity for both effective conservation strategies and detailed scientific research. The remarkable diversity of *Salvia* species in Türkiye is largely attributed to its unique geographical location at the crossroads of the Mediterranean, Euro‐Siberian, and Irano‐Turanian phytogeographical regions. In addition, the country's varied climatic conditions and complex topography create diverse ecological niches that promote species diversification and the development of endemic taxa [19].

The aim of this study was to evaluate the antioxidant capacity of extracts obtained from *Salvia heldreichiana* through DPPH analysis and total phenolic content (TPC) determination, as well as their inhibitory activities against acetylcholinesterase and tyrosinase enzymes using in vitro methods. In addition, the chemical composition of the extract was characterized by gas chromatography–mass spectrometry (GC–MS) and High‐Performance Liquid Chromatography (HPLC) analyses, and the potential interaction mechanisms of the identified major compounds with the target enzymes were investigated at the in silico level using a molecular docking approach.

## 2 Results and Discussion

### 2.1 HPLC Findings and Quantitative Phytochemical Analysis Results

The quantitative analysis of bioactive compounds in the methanolic extract of *S. heldreichiana* revealed the presence of various phenolic compounds (Table 1). Among the phenolic acids, 4‐hydroxybenzoic acid was identified as the predominant compound (1661.2 mg/L), followed by rosmarinic acid (1267.9 mg/L), coumaric acid (697.9 mg/L), and pyrogallol (621.5 mg/L). Vanillic acid (587.8 mg/L), caffeic acid (393.9 mg/L), syringic acid (344.8 mg/L), and chlorogenic acid (305.8 mg/L) were detected at moderate concentrations, whereas gallic acid, ferulic acid, resveratrol, and oleuropein were not detected within the analytical limits.

**TABLE 1:** HPLC‐based quantitative determination of bioactive compounds in *Salvia heldreichiana* extract (mg/L).

No	Compounds	*S. heldreichiana*, mg/L
Vitamin		
1	Ascorbic acid	N/D^a^
Phenolics		
2	Gallic acid	N/D
3	4‐Hydroxybenzoic acid	1661.2
4	Vanillic acid	587.8
5	Syringic acid	344.8
6	Coumaric acid	697.9
7	Caffeic acid	393.9
8	Ferulic acid	N/D
9	Rosmarinic acid	1267.9
10	Pyrogallol	621.5
11	Chlorogenic acid	305.8
12	Resveratrol	N/D
13	Oleuropein	N/D
Flavonoids		
14	Catechin	192.8
15	Epicatechin	429.9
16	Rutin	169.9
17	Myricetin	165.8
18	Quercetin	100.4
19	Apigenin	25.20
20	Cyanidin chloride	N/D
21	Hesperidin	N/D
22	Kaempferol	N/D
23	Baicalin	64.6
24	Chrysin	719.6

a N/D, not detected.

Within the flavonoid group, chrysin was present at the highest concentration (719.6 mg/L), followed by epicatechin (429.9 mg/L) and catechin (192.8 mg/L). Rutin (169.9 mg/L), myricetin (165.8 mg/L), quercetin (100.4 mg/L), baicalin (64.6 mg/L), and apigenin (25.20 mg/L) were identified at comparatively lower levels. Cyanidin chloride, hesperidin, and kaempferol were not detected. Overall, these findings indicate that *S. heldreichiana* possesses a phytochemical profile particularly rich in specific phenolic acids and selected flavonoids. Chromatograms corresponding to the identified compounds are presented in Supplementary Figure S1.

### 2.2 GC–MS Findings and Volatile Compound Profile Analysis

GC–MS analysis of the *S. heldreichiana* plant material led to the identification of 31 volatile compounds (Table 2). The dominant constituent of the volatile fraction was eucalyptol (1,8‐cineole), accounting for 34.007% of the total composition, followed by 1R‐α‐pinene (20.637%) and p‐cymene (16.065%). Notable amounts of other monoterpenes were also detected, including L‐β‐pinene (2.617%), γ‐terpinene (2.249%), cryptone (2.216%), and (+)‐2‐bornanone (2.027%). Additional compounds such as camphene (1.255%), 2‐thujene (1.935%), benzaldehyde (1.775%), estragole (1.518%), and α‐terpinene (1.514%) were present in moderate proportions. Among the sesquiterpenes, germacrene D (0.222%) and trans‐calamenene (0.250%) were identified, while the diterpene abietatriene was detected at 0.290%. Overall, the volatile profile was characterized by a predominance of monoterpene hydrocarbons and oxygenated monoterpenes, with eucalyptol and α‐pinene representing the major constituents of the essential oil composition. Chromatograms corresponding to the identified compounds are presented in Supplementary Figure S2.

**TABLE 2:** GC–MS analysis of the volatile profile of *Salvia heldreichiana* plant material.

No	RT (min)	RI	Name of the compound	Content, %
1	6.059	797	Hexanal	0.707
2	7.630	846	2‐Hexenal, (E)	1.792
3	8.969	887	Styrene	1.62
4	10.371	923	alpha.‐Phellandrene	0.596
5	10.650	930	1R‐α‐Pinene	20.637
6	11.259	944	Camphene	1.255
7	11.491	950	2‐Thujene	1.935
8	11.728	955	Benzaldehyde	1.775
9	12.316	969	α‐Sabinene	0.344
10	12.446	973	L‐β‐Pinene	2.617
11	13.644	1001	3‐Carene	0.630
12	14.202	1013	α‐Terpinene	1.514
13	14.565	1021	p‐Cymene	16.065
14	14.867	1028	Eucalyptol	34.007
15	16.503	1063	gamma.‐Terpinene	2.249
17	19.242	1123	alpha.‐Campholenal	0.153
18	19.826	1135	trans‐Pinocarveole	0.543
19	20.094	1141	(+)‐2‐Bornanone	2.027
20	20.948	1159	Pinocarvone	0.348
21	21.066	1162	endo‐Borneol	1.066
22	22.015	1183	Crypton	2.216
23	22.493	1193	(1R)‐(‐)‐Myrtenal	0.323
24	22.568	1195	Estragole	1.518
25	23.088	1206	Levoverbenone	0.55
26	24.439	1236	p‐Cumic aldehyde	1.648
27	26.012	1271	3‐p‐Menthen‐7‐al	0.261
28	27.127	1296	Carvacrol	0.250
29	36.052	1509	Germacrene D	0.222
30	36.428	1517	trans‐Calamenene	0.250
31	49.619	2036	Abitatriene	0.290

### 2.3 Antioxidant Activity and Enzyme Inhibition

The antioxidant activity of the methanolic extract of *S. heldreichiana* was evaluated using the DPPH radical scavenging assay, yielding an IC_50_ value of 0.26 ± 0.03 mg/mL. The TPC was determined to be 498.8 ± 82.4 mg GAE/g extract (Table 3). These findings indicate that *S. heldreichiana* exhibits considerable antioxidant capacity and possesses a phytochemical profile rich in phenolic compounds. The results further suggest a potential correlation between phenolic content and free radical scavenging activity.

**TABLE 3:** Antioxidant activity and total phenolic content of *Salvia heldreichiana* extract.

	DPPH assay (IC_50_ mg/mL)	Total phenolic content (mg GAE/g extract)
*S. heldreichiana* extract	0.26 ± 0.03	498.8 ± 82.4

The inhibitory effects of the methanolic extract of *S. heldreichiana* on AChE and tyrosinase (Tyr) were evaluated and expressed as IC_50_ values (mg/mL). The extract exhibited an IC_50_ value of 7.97 ± 0.54 mg/mL against AChE and 8.69 ± 1.35 mg/mL against tyrosinase. For comparison, the positive control galantamine showed an IC_50_ value of 1.04 ± 0.48 mg/mL for AChE inhibition, while kojic acid, used as the reference inhibitor for tyrosinase, demonstrated an IC_50_ value of 0.32 ± 0.04 mg/mL (Table 4).

**TABLE 4:** Enzyme inhibitory activity of *Salvia heldreichiana* extract against AChE and tyrosinase.

	AChE inhibitory activity (IC_50_ mg/mL)	Tyrosinase inhibitory activity (IC_50_ mg/mL)
*S. heldreichiana* extract	7.97 ± 0.54	8.69 ± 1.35
Galantamine	1.04 ± 0.48	—
Kojic acid	—	0.32 ± 0.04

These findings indicate that *S. heldreichiana* extract exerts measurable inhibitory activity against both enzymes. Although its potency is lower than that of the standard inhibitors, the observed activity is noteworthy for a crude plant extract and suggests the presence of bioactive constituents contributing to enzyme inhibition.

In the present study, the phytochemical composition, antioxidant capacity, and enzyme inhibitory potential of the methanolic extract and volatile fraction of *S. heldreichiana* were comprehensively evaluated and interpreted in light of all previously reported literature. HPLC analysis revealed that 4‐hydroxybenzoic acid (1661.2 mg/L), rosmarinic acid (1267.9 mg/L), p‐coumaric acid (697.9 mg/L), pyrogallol (621.5 mg/L), and vanillic acid (587.8 mg/L) were the predominant phenolic constituents, while chrysin (719.6 mg/L) and epicatechin (429.9 mg/L) were present at remarkably high levels among flavonoids. The prominent presence of rosmarinic acid is consistent with the findings of Yılmaz et al. (2023), who reported rosmarinic acid as the major phenolic compound (29.376 mg/g extract), supporting the phenolic richness of the species [20]. Similarly, Bardakcı et al. (2019) documented the occurrence of caffeic acid and rosmarinic acid across different fractions, which aligns with our data. However, unlike some earlier reports in which catechin, epicatechin, and rutin were not detected, our study identified these compounds, suggesting that solvent polarity, geographic origin, and analytical methodology significantly influence the phenolic profile [21].

GC–MS analysis of the volatile fraction demonstrated that eucalyptol (1,8‐cineole) (34.007%), 1R‐α‐pinene (20.637%), and p‐cymene (16.065%) were the dominant constituents. This general monoterpene‐dominant profile is in agreement with previous reports [19, 21, 22, 23, 24] all of whom described α‐pinene and oxygenated monoterpenes as principal components of *S. heldreichiana* essential oil. Nevertheless, the markedly high proportion of 1,8‐cineole observed in the present study indicates a cineole‐dominant chemotype, highlighting potential chemotypic variability within the species.

The antioxidant activity determined by the DPPH assay (IC_50_ = 0.26 ± 0.03 mg/mL) is highly comparable to the value reported by Yılmaz et al. (2023) (IC_50_ = 235.5 µg/mL), confirming the strong radical scavenging capacity of the species [20]. These findings are further supported by the pronounced antioxidant activities previously documented by Bardakcı et al. (2019) and Şenol et al. (2010), particularly in polar fractions rich in phenolics. The high TPC measured in our study (498.8 ± 82.4 mg GAE/g extract) reinforces the established correlation between phenolic abundance and antioxidant potential [21, 25].

Regarding enzyme inhibition, the extract exhibited measurable inhibitory activity against AChE (IC_50_ = 7.97 ± 0.54 mg/mL) and tyrosinase (IC_50_ = 8.69 ± 1.35 mg/mL). Although these values indicate lower potency compared to standard inhibitors, they are consistent with the moderate to weak enzyme inhibitory activities reported by Yılmaz et al. (2023) and Şenol et al. (2010). The observed activity may be attributed to the synergistic contribution of rosmarinic acid and flavonoids such as chrysin and epicatechin, which are known to possess enzyme‐modulating properties. Furthermore, the abundance of monoterpenes such as 1,8‐cineole and α‐pinene may also contribute to the biological profile of the species, consistent with the antibacterial activity reported by Yılmaz et al. (2024) and the anxiolytic effects demonstrated by Daştan et al. (2025) [26, 27].

### 2.4 CAVER Tunnel and Molecular Docking Analysis

**FIGURE 1:**
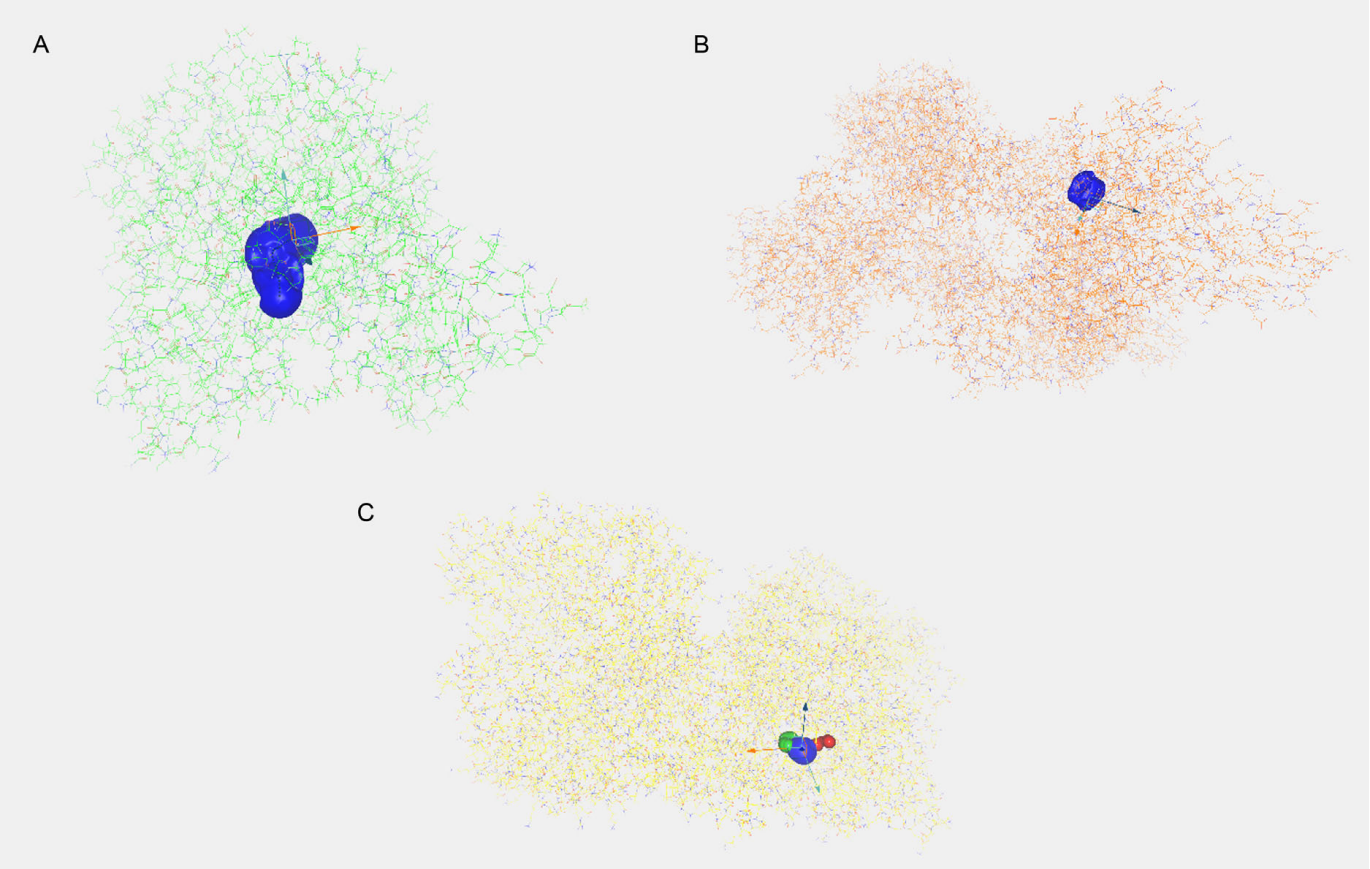
Structural visualization of continuous ligand access tunnels from the active site to the protein surface in 1ACJ (A), 2Y9X (B), and 3NVY (C) identified by CAVER.

The CAVER analysis identified continuous and geometrically feasible primary tunnels extending from the active site to the protein surface in all investigated protein structures (Table 5). In the 1ACJ structure, the primary tunnel exhibited an average length of 10.71 Å and an average throughput value of 0.749, indicating a structurally permissive pathway for ligand transport (Figure 1A). In contrast, the 2Y9X protein displayed a markedly shorter tunnel (Avg_L = 1.93 Å) with a larger average bottleneck radius (Avg_BR = 1.86 Å), resulting in the highest average throughput value (0.932) among the analyzed structures. This finding suggests that ligands may traverse the tunnel in 2Y9X with minimal geometric restriction and enhanced transport efficiency (Figure 1B).

**TABLE 5:** Geometric and functional parameters of the primary tunnels identified by CAVER analysis.

Protein, PDB	Avg_BR, Å	Avg_L, Å	Avg_C	Avg_throughput
1ACJ	1.56	10.71	1.133	0.749
2Y9X	1.86	1.93	1.157	0.932
3NVY	1.81	5.92	1.086	0.820

*Note:* Avg_BR (Å), average bottleneck radius of the tunnel; Avg_L (Å), average tunnel length; Avg_C, average curvature of the tunnel; Avg_throughput, average throughput representing the tunnel's ligand transport capacity.

For the 3NVY structure, the identified primary tunnel showed intermediate geometric characteristics, with an average length of 5.92 Å and an average throughput of 0.820, reflecting a balanced tunnel architecture capable of supporting ligand migration (Figure 1C). Overall, the combined evaluation of average bottleneck radius, tunnel length, and throughput values demonstrates the presence of structurally accessible channels in all proteins analyzed. These results provide a solid geometric basis for subsequent docking and binding path analyses, supporting the feasibility of ligand migration from the active site to the protein surface.

**FIGURE 2:**
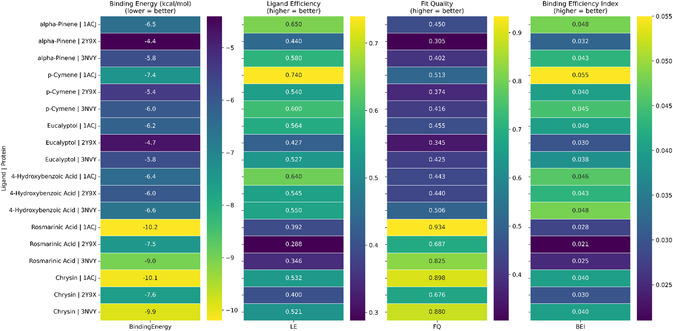
Comparative molecular docking parameters (Δ*G*, LE, FQ, and BEI) of selected major compounds against 1ACJ, 2Y9X, and 3NVY protein targets.

When the molecular docking results presented in Figure 2 were examined in detail, the binding free energies (Δ*G*) of α‐pinene against 1ACJ, 2Y9X, and 3NVY were calculated as −6.5, −4.4, and −5.8 kcal/mol, respectively. The corresponding ligand efficiency (LE) values were 0.650, 0.440, and 0.580; fit quality (FQ) values were 0.450, 0.305, and 0.402; and binding efficiency index (BEI) values were 0.048, 0.032, and 0.043. For p‐cymene, the Δ*G* values obtained for 1ACJ, 2Y9X, and 3NVY were −7.4, −5.4, and −6.0 kcal/mol, with LE values of 0.740, 0.540, and 0.600; FQ values of 0.513, 0.374, and 0.416; and BEI values of 0.055, 0.040, and 0.045, respectively. Eucalyptol exhibited binding energies of −6.2, −4.7, and −5.8 kcal/mol toward 1ACJ, 2Y9X, and 3NVY, with corresponding LE values of 0.564, 0.427, and 0.527; FQ values of 0.455, 0.345, and 0.425; and BEI values of 0.040, 0.030, and 0.038.

Similarly, 4‐hydroxybenzoic acid showed Δ*G* values of −6.4, −6.0, and −6.6 kcal/mol against 1ACJ, 2Y9X, and 3NVY, respectively, with LE values of 0.640, 0.545, and 0.550; FQ values of 0.443, 0.440, and 0.506; and BEI values of 0.046, 0.043, and 0.048. Rosmarinic acid demonstrated binding free energies of −10.2, −7.5, and −9.0 kcal/mol toward 1ACJ, 2Y9X, and 3NVY, respectively, accompanied by LE values of 0.392, 0.288, and 0.346; FQ values of 0.934, 0.687, and 0.825; and BEI values of 0.028, 0.021, and 0.025. Chrysin exhibited Δ*G* values of −10.1, −7.6, and −9.9 kcal/mol against 1ACJ, 2Y9X, and 3NVY, with corresponding LE values of 0.532, 0.400, and 0.521; FQ values of 0.898, 0.676, and 0.880; and BEI values of 0.040, 0.030, and 0.040.

Among all evaluated complexes, rosmarinic acid displayed the strongest binding affinity toward 1ACJ (−10.2 kcal/mol). For 2Y9X (tyrosinase, PPO3), the lowest Δ*G* value was obtained for chrysin (−7.6 kcal/mol), while for 3NVY (xanthine oxidase), chrysin again showed the most favorable binding energy (−9.9 kcal/mol). These findings indicate that rosmarinic acid is the most potent ligand for 1ACJ, whereas chrysin exhibits the highest binding affinity toward 2Y9X and 3NVY.

**FIGURE 3:**
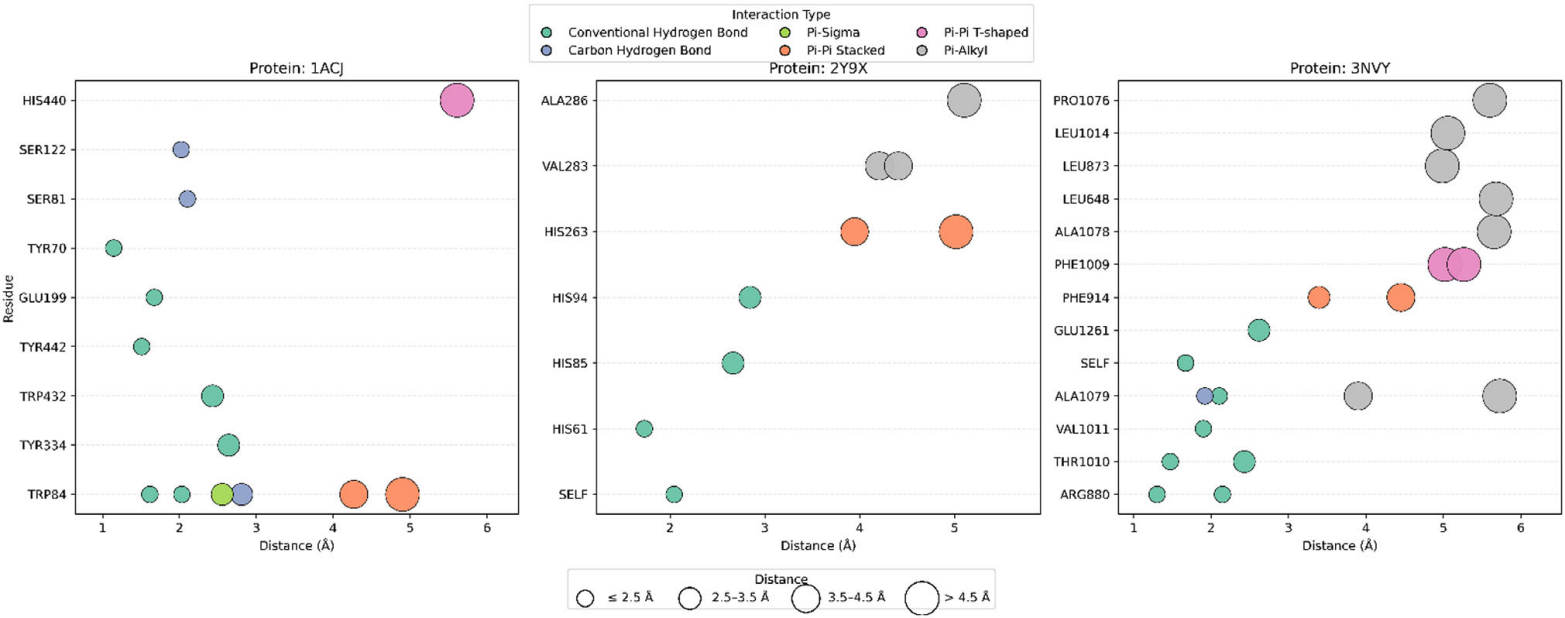
Comparative residue interaction profiles of the major ligand with 1ACJ, 2Y9X, and 3NVY.

In Figure 3, the bubble plot illustrates the residue‐level interaction profile of the major ligand with 1ACJ, 2Y9X, and 3NVY proteins in terms of interaction type and binding distance (Å). In the graph, the *x*‐axis represents interaction distance (Å), whereas the *y*‐axis indicates the interacting amino acid residues. Bubble size reflects distance range, and color coding denotes the type of interaction, including conventional hydrogen bonds, carbon hydrogen bonds, π–sigma interactions, π–π stacking, π–π T‐shaped interactions, and π–alkyl contacts.

**FIGURE 4:**
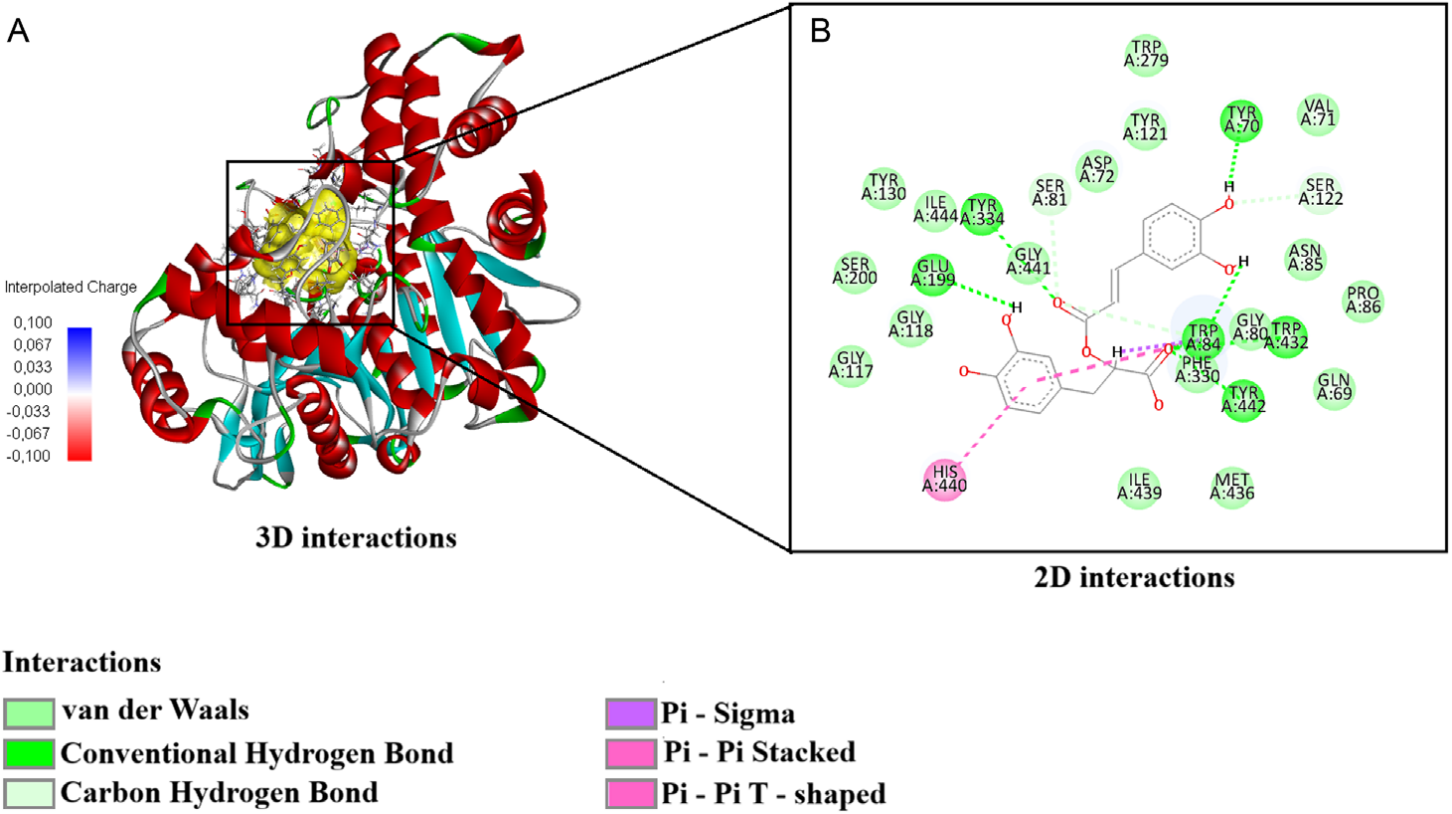
Three‐dimensional (A) and two‐dimensional (B) interaction analysis of rosmarinic acid in 1ACJ.

For 1ACJ, interactions were observed with residues TRP84, TYR334, TRP432, TYR70, GLU199, SER81, SER122, and HIS440. The interaction distances ranged approximately between 1.8 and 5.5 Å. Conventional hydrogen bonds were primarily detected within the 2.0–3.0 Å range, π‐based interactions were concentrated around 3.0–4.0 Å, and longer distance hydrophobic contacts were observed beyond 4.0 Å (Figure 4).

**FIGURE 5:**
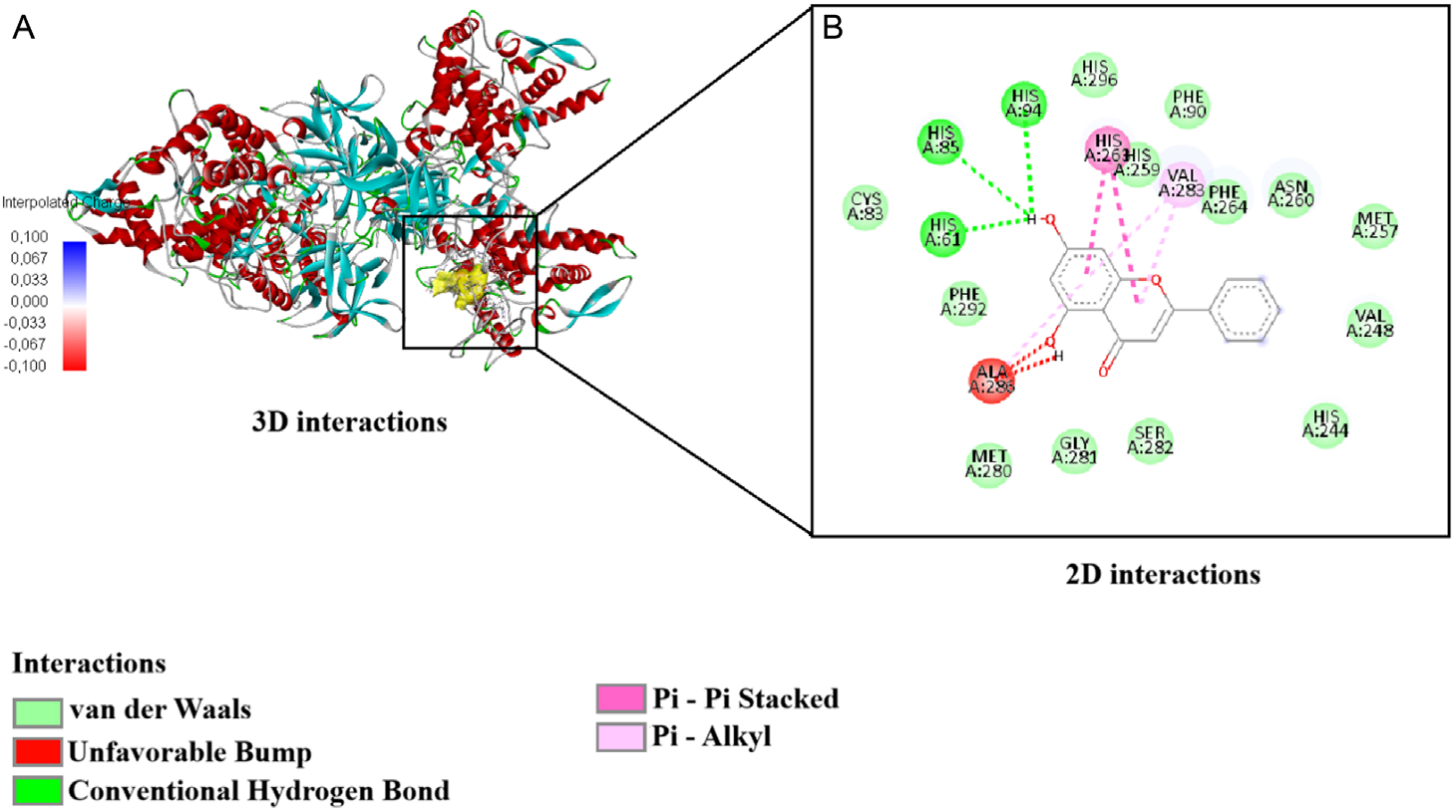
Three‐dimensional (A) and two‐dimensional (B) interaction analysis of chrysin acid in 2Y9X.

In the 2Y9X protein, the ligand interacted mainly with HIS61, HIS85, HIS94, HIS263, VAL283, and ALA286. The interaction distances varied between approximately 1.9 and 5.0 Å. Hydrogen bonds were predominantly distributed within 2.0–3.0 Å, whereas π–π stacking and π–alkyl interactions were observed mainly within the 3.5–5.0 Å range (Figure 5).

**FIGURE 6:**
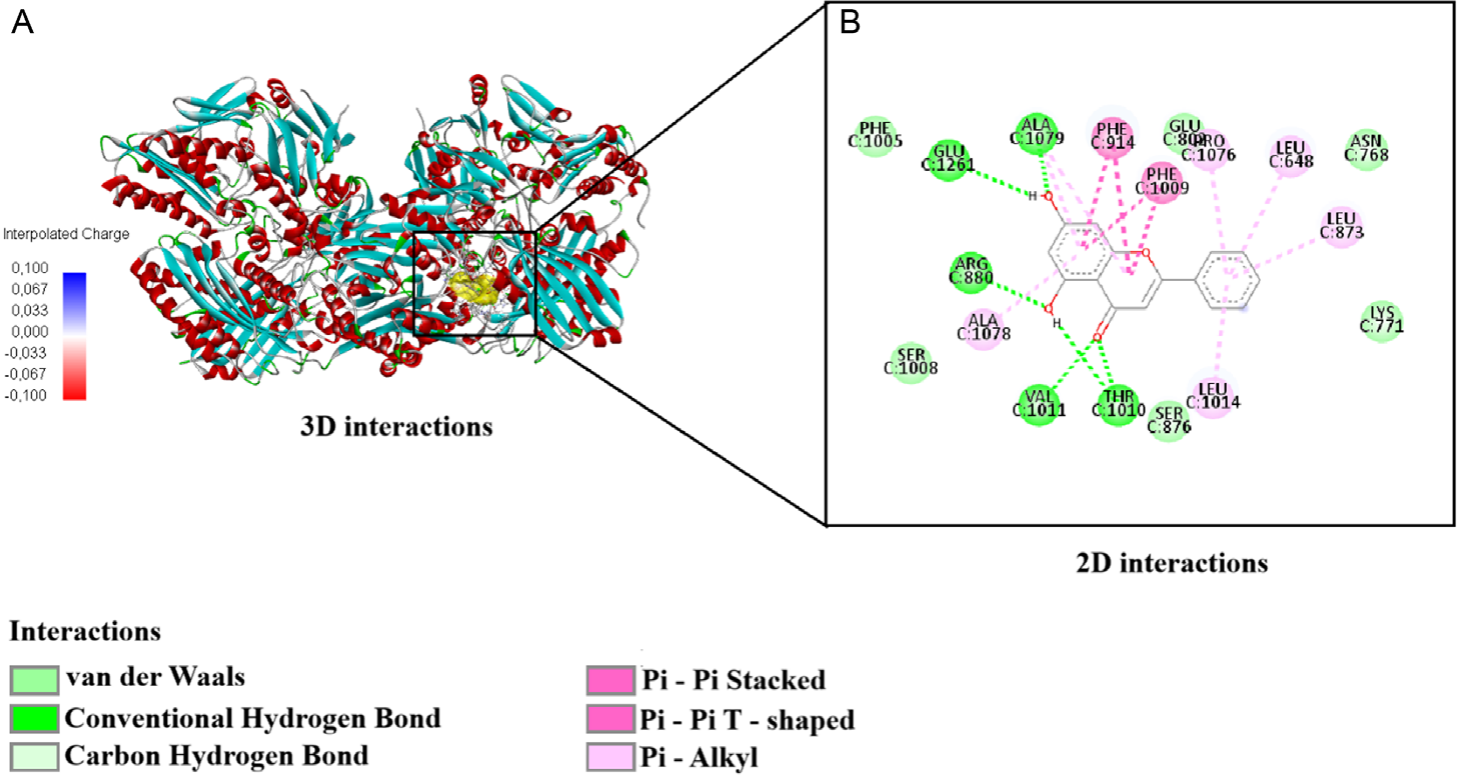
Three‐dimensional (A) and two‐dimensional (B) interaction analysis of chrysin acid in 3NVY.

For the 3NVY protein, interactions were identified with ARG880, THR1010, VAL1011, ALA1079, GLU1261, PHE914, PHE1009, ALA1078, LEU648, LEU873, LEU1014, and PRO1076. The interaction distances ranged from approximately 1.6 to 6.0 Å. Short‐range hydrogen bonds were observed within 1.6–2.5 Å, carbon hydrogen bonds within 2.5–3.5 Å, π–π T‐shaped interactions with aromatic residues (notably PHE914 and PHE1009) around 4.5–5.5 Å, and hydrophobic π–alkyl contacts predominantly within 3.5–6.0 Å (Figure 6).

Overall, the bubble plot in Figure 3 demonstrates that, across all three proteins, the ligand forms multiple non‐covalent interactions spanning short‐, medium‐, and long‐range distances, indicating a structurally diverse interaction network within the active site regions.

## 3 Conclusion

This study demonstrates that *S. heldreichiana* possesses a phenolic‐rich phytochemical profile and notable biological activity supported by both experimental and computational findings. HPLC analysis revealed high levels of 4‐hydroxybenzoic acid, rosmarinic acid, p‐coumaric acid, pyrogallol, and chrysin, which likely contribute to the strong antioxidant activity observed (DPPH IC_50_ = 0.26 ± 0.03 mg/mL; TPC = 498.8 ± 82.4 mg GAE/g extract). GC–MS analysis confirmed a monoterpene‐dominated volatile profile characterized by eucalyptol, α‐pinene, and p‐cymene. The extract exhibited measurable inhibitory activity against AChE and tyrosinase, suggesting potential neuroprotective and dermatological relevance. CAVER analysis verified structurally accessible tunnels in all target proteins, supporting ligand transport feasibility. Molecular docking identified rosmarinic acid and chrysin as the strongest binders, particularly toward 1ACJ and 3NVY, with favorable binding energies and interaction profiles. Overall, the concordance between phytochemical composition, in vitro bioactivity, and in silico interaction analyses highlights *S. heldreichiana* as a promising natural source of bioactive compounds warranting further pharmacological investigation.

## 4 Experimental Section

### 4.1 Collection, Identification, and Extraction of Plant Material

The specimens of *S. heldreichiana* Boiss. ex Benth. used in this study were collected from their natural habitat on 05.07.2024 in the Gülek area (roadside), Tarsus district, Mersin Province, Türkiye, at an altitude of 1250 m (C4 grid; S. Doğu 3113). For comparative purposes, *Salvia hypargeia* Fisch. & C.A. Mey. specimens were collected on 07.06.2024 from forest clearings in Bal Forest, along the Konya–Beyşehir road, Meram district, Konya Province, Türkiye, at 1200 m altitude (C4 grid; S. Doğu 3112). All plant materials were harvested from their natural populations. The collected specimens were dried according to standard herbarium techniques and deposited in the Herbarium of the Medical and Cosmetic Plants Research Center, Necmettin Erbakan University. Taxonomic identification was performed by Dr. Süleyman Doğu.

For extraction, the plant material of *S. heldreichiana* was carefully cleaned to remove foreign matter. The samples were dried in a ventilated oven at 40°C for 48 h to preserve thermolabile bioactive constituents and subsequently ground into a fine powder to ensure homogeneity. Approximately 30 g of the powdered material was extracted with methanol at a ratio of 1:10 (w/v). The extraction process was carried out by static maceration for 72 h at room temperature in the dark to prevent degradation of light‐sensitive compounds. Following filtration, the solvent was removed under reduced pressure at 40°C using a rotary evaporator. The concentrated methanolic extract was stored in airtight containers at 4°C until further analysis [28].

### 4.2 Quantitative Determination of Phenolic Compounds by High‐Performance Liquid Chromatography with Diode Array Detection (HPLC‐DAD)

The quantitative analysis of phenolic compounds in the *S. heldreichiana* extract was performed using an HPLC‐DAD system. External standard calibration was applied, and six‐point calibration curves were established for each reference compound within the concentration range of 25–300 µg/mL.

In the first chromatographic method, L‐ascorbic acid, gallic acid, 3,4‐dihydroxybenzoic acid, (+)‐catechin, (–)‐epicatechin, trans‐caffeic acid, vanillic acid, rutin, p‐coumaric acid, ferulic acid, rosmarinic acid, myricetin, quercetin, and apigenin were analyzed. The mobile phase consisted of acetonitrile (A) and 1.5% aqueous acetic acid (B), with a linear gradient elution from 15% to 40% A over 29 min. Detection was carried out at 250, 270, and 320 nm.

The second analytical method targeted pyrogallol, chlorogenic acid, syringic acid, cyanidin chloride, resveratrol, oleuropein, hesperidin, kaempferol, baicalein, and chrysin. In this protocol, the mobile phase was composed of methanol (A) and 1.5% aqueous acetic acid (B), with a gradient program progressing from 10% to 90% A over 53 min. Detection wavelengths were set at 280, 290, 320, 370, and 535 nm.

For both methods, an ACE 5 C18 reversed‐phase column (250 × 4.6 mm, 5 µm) was used. The flow rate was maintained at 0.7 mL/min, the injection volume was 10 µL, and the column temperature was controlled at 35°C [29].

### 4.3 Determination of Volatile Compounds by GC–MS Using the SPME Technique

The volatile profile of *S. heldreichiana* plant material was analyzed by GC–MS following solid‐phase microextraction (SPME). The SPME fiber was preconditioned according to the manufacturer's instructions. Finely ground and homogenized plant samples were transferred into 20‐mL glass vials sealed with PTFE/silicone septa, filling approximately one‐third of the vial volume. The vials were equilibrated at 45°C for 15 min to ensure thermal stabilization. Subsequently, the SPME fiber was exposed to the headspace of the samples for 40 min to allow adsorption of volatile constituents. After extraction, the fiber was inserted into the GC injector port, where thermal desorption was performed at 250°C for 20 min. Chromatographic analyses were carried out using an Agilent gas chromatograph equipped with an HP‐5 MS ultra inert capillary column (30 m × 0.25 mm i.d., 0.25 µm film thickness). High‐purity helium (≥99.99%) was used as the carrier gas at a constant flow rate of 1.0 mL/min. Injections were performed in splitless mode. The oven temperature program was set as follows: initial temperature at 50°C (held for 2 min), increased to 150°C at 2.5°C/min (held for 5 min), and finally raised to 250°C at 6.5°C/min (held for 1 min). The mass spectrometer operated in electron ionization (EI) mode at 70 eV, scanning a mass range of 35–500 m/z in full scan mode [30].

### 4.4 2,2‐Diphenyl‐1‐Picrylhydrazyl (DPPH) Radical Scavenging Assay

The free radical scavenging activity of the *S. heldreichiana* extracts was evaluated using the DPPH assay, and the results were compared with the synthetic antioxidant butylated hydroxytoluene (BHT) as a reference standard. Briefly, 50 µL of extract solutions at different concentrations was mixed with 50 µL of 0.1 mM DPPH solution in a 96‐well microplate. The reaction mixture was incubated in the dark for 30 min at room temperature to prevent photodegradation [31].

After incubation, absorbance values were recorded at 517 nm using a microplate reader (Thermo Scientific Varioskan Flash), with a blank solution serving as the reference. The percentage of DPPH radical scavenging activity was calculated according to the following equation:

$$\text{DPPH scavenging activity}\left(\text{\% inhibition}\right)=\left[\frac{\left({\text{Absorbance}}_{\text{control}}\right)-\left({\text{Absorbance}}_{\text{sample}}\right)}{{\text{Absorbance}}_{\text{control}}}\right]\times 100$$

A concentration–inhibition curve was constructed, and the IC_50_ value, defined as the extract concentration required to reduce the initial DPPH concentration by 50%, was calculated by linear regression analysis [32].

### 4.5 Total Phenolic Content (TPC)

The TPC of the *S. heldreichiana* extracts was determined using a modified Folin–Ciocalteu colorimetric method, as originally described by Singleton and Rossi (1965). Briefly, 10 µL of extract solution (1 mg/mL) was mixed with 20 µL of Folin–Ciocalteu reagent and allowed to react for 3 min at room temperature. Subsequently, 100 µL of 2% sodium carbonate solution was added, and the mixture was incubated for 1 h to allow color development [33].

Absorbance was measured at 760 nm using a microplate reader (Thermo Scientific Varioskan Flash). The results were expressed as milligrams of gallic acid equivalents per gram of extract (mg GAE/g extract). All analyses were performed in triplicate.

### 4.6 Acetylcholinesterase (AChE) Inhibitory Activity

The determination of acetylcholinesterase inhibitory activity was performed using an adapted 96‐well microplate assay, based on Ellman's method [34, 35, 36]. The samples were diluted to 10 mg/mL in 50 mM Tris buffer at pH 8.0. The samples were subjected to a twofold dilution process, employing a Tris buffer solution as the diluent. The addition of sample solutions to a 96‐well microplate was followed by the subsequent addition of 25 μL of 0.22 U/mL AChE, 3 mM 5,5′‐dithiobis[2‐nitrobenzoic acid] (DTNB) (125 μL), and 50 μL of Tris buffer. Subsequently, the microplate was pre‐incubated for 5 min at 25°C in the dark. The reaction was initiated by the addition of 25 μL of substrate, prepared as 1.5 mM acetylthiocholine iodide (ATCI). Galantamine was utilized as positive control. Enzyme activity was calculated as a percentage of the velocity of the test sample in relation to that of the non‐treated control. The inhibitory activity was calculated as follows:

$$\%\text{inhibition}=\left[\frac{\mathrm{mean}\;\text{velocity}\;\mathrm{of}\;\text{control}-\mathrm{mean}\;\text{velocity}\;\mathrm{of}\;\text{sample}}{\mathrm{mean}\;\text{velocity}\;\mathrm{of}\;\text{control}}\right]\times 100$$

The IC_50_ values (concentrations at which 50% of enzyme activity is inhibited) were determined using linear regression analysis. All samples were analyzed in triplicate.

### 4.7 Tyrosinase (Tyr) Inhibitory Activity

The tyrosinase inhibitory activity of the samples was determined by employing the modified dopachrome method, utilizing L‐DOPA as the substrate, with minor modifications [37, 38]. The samples were diluted to 10 mg/mL in 50 mM phosphate buffer at pH 6.8. The samples were subjected to a twofold dilution process, employing a phosphate buffer solution as the diluent. The addition of sample solutions (25 μL) to a 96‐well microplate was followed by the addition of 25 μL of enzyme solution (100 U/mL). Subsequently, the microplate was pre‐incubated for 5 min at 25°C in the dark. The reaction was initiated by the addition of 100 μL of 2 mM L‐DOPA (3,4‐dihydroxy‐L‐phenylalanine) substrate. Kojic acid was utilized as a positive control. Enzyme activity was calculated as a percentage of the velocity of the test sample in relation to that of the non‐treated control. The inhibitory activity was calculated as follows

$$\%\text{inhibition}=\left[\frac{\mathrm{mean}\;\text{velocity}\;\mathrm{of}\;\text{control}-\mathrm{mean}\;\text{velocity}\;\mathrm{of}\;\text{sample}}{\mathrm{mean}\;\text{velocity}\;\mathrm{of}\;\text{control}}\right]\times 100$$

The IC_50_ values (concentrations at which 50% of enzyme activity is inhibited) were determined using linear regression analysis. All samples were analyzed in triplicate.

### 4.8 CAVER Analysis

Tunnel analysis was performed using CAVER Analyst to identify potential ligand transport pathways within the protein structures. Crystal structures obtained from the Protein Data Bank (PDB) were used as input, and all proteins were preprocessed by removing crystallographic water molecules, ions, and other non‐essential heteroatoms prior to analysis. For each protein, the starting point was defined using preselected Cartesian coordinates corresponding to the active site region.

Tunnel calculations were carried out using default and commonly accepted CAVER parameters reported in the literature. Specifically, the minimum probe radius was set to 0.9 Å, shell depth to 4, shell radius to 3, and the clustering threshold to 3.5. All analyses were conducted on a single static protein structure (single snapshot), without incorporating molecular dynamics simulations.

Following tunnel detection, all identified tunnels were evaluated based on their geometric and functional descriptors, including average bottleneck radius (Avg_BR), average tunnel length (Avg_L), average curvature (Avg_C), and average throughput (Avg_throughput). In cases where multiple tunnels were detected within a single protein structure, only the tunnel exhibiting the highest Avg_throughput value was considered the primary tunnel and selected for quantitative analysis, while alternative tunnels were regarded as secondary and excluded from further evaluation.

Three‐dimensional visualization of the identified primary tunnels was performed within the CAVER Analyst environment to confirm their continuity and spatial orientation relative to the protein structure. The resulting tunnel parameters were used to assess the geometric feasibility of ligand migration from the active site toward the protein surface and to provide a structural basis for subsequent docking and binding path analyses [39].

### 4.9 Molecular Docking

Molecular docking studies were conducted to evaluate the binding affinity of the major compound identified in the plant extract toward selected target enzymes. The three‐dimensional crystal structures of the proteins were retrieved from the PDB. In this context, PDB ID: 1ACJ was used for acetylcholinesterase, PDB ID: 2Y9X for *Agaricus bisporus* tyrosinase (PPO3), and PDB ID: 3NVY for bovine xanthine oxidase.

Prior to docking, protein preparation was performed by removing water molecules not directly involved in the active site. The binding pocket was verified based on the coordinates of the co‐crystallized ligand and known inhibitors. Missing hydrogen atoms were added, appropriate protonation states were assigned considering physiological pH conditions, and Gasteiger partial charges were calculated. The prepared protein structures were saved in a format suitable for docking analysis.

The three‐dimensional structure of the major ligand was subjected to energy minimization. Geometric optimization was applied while preserving torsional flexibility by allowing rotatable bonds to remain active, thereby enabling conformational adaptability during docking.

Docking simulations were carried out using the AutoDock Vina algorithm [40]. The docking procedure focused on the experimentally defined active site regions, and multiple ligand conformations were evaluated. The binding pose with the lowest binding free energy (Δ*G*, kcal/mol) was selected as the most favorable binding mode.

The resulting protein–ligand complexes were further analyzed using structural visualization tools. Interactions were characterized in terms of conventional hydrogen bonds, carbon–hydrogen bonds, π–π stacking, π–alkyl, and alkyl interactions. Bond distances were measured, and contacts with key active site amino acid residues were examined in detail [41].

## Author Contributions

**Erdi Can Aytar**: writing – review and editing, methodology, supervisor, formal analysis, conceptualization, investigation. **Esin Çolak**: writing – review and editing, writing – original draft, methodology, investigation, data curation. **Kaan Bedirhan Kahveci**: writing – review and editing, visualization, investigation, conceptualization. **Süleyman Doğu**: plant material collection and supply, writing – review and editing, investigation. **Abidin Gümrükçüoğlu**: writing – review and editing, visualization, investigation, conceptualization, validation. **Betül Aydın**: review and editing, methodology, formal analysis, investigation, data curation.

## Declaration of Generative Ai and Ai‐Assisted Technologies in the Writing Process

During the preparation of this work, the author(s) used ChatGPT (OpenAI) to improve the clarity and language of the manuscript.

## Conflicts of Interest

The authors declare no conflicts of interest.

## Supporting information

Supplementary Material

## Data Availability

The data that support the findings of this study are available from the corresponding author upon reasonable request.
