# Supplementary material for: Evaluation of the Phytochemical Composition, Antioxidant Activity, and Enzyme Inhibitory Potential of Salvia heldreichiana Within the Framework of Molecular Docking and CAVER Tunnel Analysis
Source: ChemistryOpen. 2026 Jun 1;15(6):e70240. doi: 10.1002/open.70240 (PMC13239546; doi:10.1002/open.70240)
Supplement: Supplementary file 1 — Supplementary Material [file OPEN-15-e70240-s001.pdf]

Evaluation of the Phytochemical Composition, Antioxidant Activity, and Enzyme Inhibitory Potential of *Salvia heldreichiana* within the Framework of Molecular Docking and CAVER Tunnel Analysis

Erdi Can Aytar[1]*, Esin Çolak[2], Kaan Bedirhan Kahveci[2], Abidin Gümrükçüoğlu[3], Süleyman Doğu[4,5], Betül Aydın[2]

[1]Usak University, Faculty of Agriculture, Department of Horticulture, Usak, Türkiye

[2] Gazi University, Faculty of Science, Department of Biology, Ankara-Türkiye

[3]Artvin Çoruh University, Medicinal-Aromatic Plants Application and Research Center, Artvin, Türkiye

[4]Necmettin Erbakan University, Faculty of Medicine, Department of Medical Biochemistry, 42090, Konya, Türkiye

[5]Necmettin Erbakan University, Meram Vocational School Konya, Turkey

*** Correspondence to:** Dr. Erdi Can Aytar

erdicanaytar@gmail.com

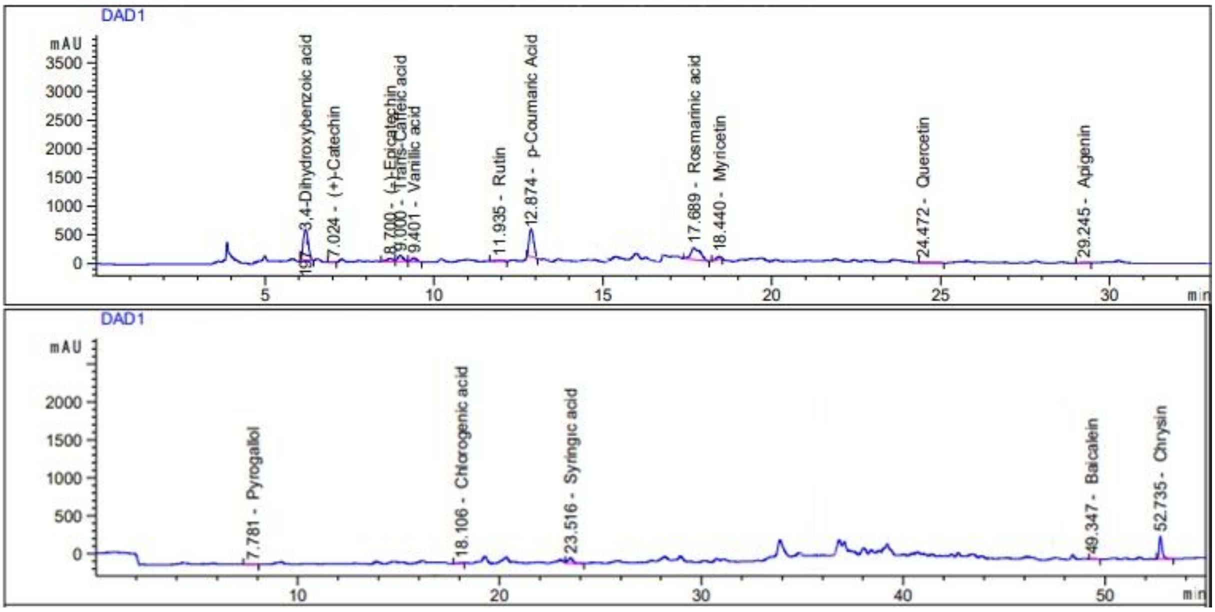

Figure S1. HPLC chromatograms of the methanolic extract of *S. heldreichiana*

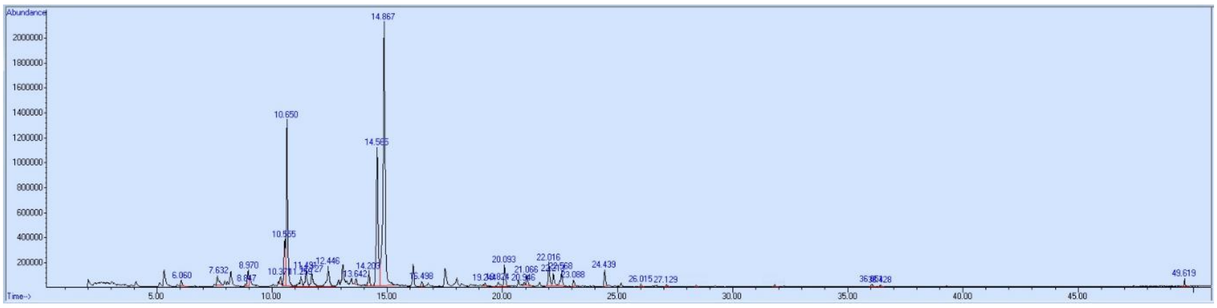

Figure S2. GC–MS chromatograms of the methanolic extract of *S. heldreichiana*.
